# Temporal extracellular vesicle protein changes following intraarticular treatment with integrin α10β1-selected mesenchymal stem cells in equine osteoarthritis

**DOI:** 10.3389/fvets.2022.1057667

**Published:** 2022-11-24

**Authors:** Emily J. Clarke, Emily Johnson, Eva Caamaño Gutierrez, Camilla Andersen, Lise C. Berg, Rosalind E. Jenkins, Casper Lindegaard, Kristina Uvebrant, Evy Lundgren-Åkerlund, Agnieszka Turlo, Victoria James, Stine Jacobsen, Mandy J. Peffers

**Affiliations:** ^1^Institute of Life Course and Medical Sciences, University of Liverpool, Liverpool, United Kingdom; ^2^Computational Biology Facility, Liverpool Shared Research Facilities, Faculty of Health and Life Sciences, University of Liverpool, Liverpool, United Kingdom; ^3^Department of Veterinary Clinical Sciences, University of Copenhagen, Copenhagen, Denmark; ^4^Department of Pharmacology and Therapeutics, Institute of Systems, Molecular and Integrative Biology, Centre for Drug Safety Science Bioanalytical Facility, Liverpool Shared Research Facilities, University of Liverpool, Liverpool, United Kingdom; ^5^Xintela AB, Lund, Sweden; ^6^School of Veterinary Medicine and Science, University of Nottingham, Nottingham, United Kingdom

**Keywords:** equine, osteoarthritis, extracellular vesicles, biologics, MSC therapy

## Abstract

**Introduction:**

Equine osteoarthritis (OA) is a heterogeneous, degenerative disease of the musculoskeletal system with multifactorial causation, characterized by a joint metabolic imbalance. Extracellular vesicles are nanoparticles involved in intracellular communication. Mesenchymal stem cell (MSC) therapy is a form of regenerative medicine that utilizes their properties to repair damaged tissues. Despite its wide use in veterinary practice, the exact mechanism of action of MSCs is not fully understood. The aim of this study was to determine the synovial fluid extracellular vesicle protein cargo following integrin α10β1-selected mesenchymal stem cell (integrin α10-MSC) treatment in an experimental model of equine osteoarthritis with longitudinal sampling.

**Methods:**

Adipose tissue derived, integrin α10-MSCs were injected intraarticularly in six horses 18 days after experimental induction of OA. Synovial fluid samples were collected at day 0, 18, 21, 28, 35, and 70. Synovial fluid was processed and extracellular vesicles were isolated and characterized. Extracellular vesicle cargo was then analyzed using data independent acquisition mass spectrometry proteomics.

**Results:**

A total of 442 proteins were identified across all samples, with 48 proteins differentially expressed (FDR ≤ 0.05) between sham-operated control joint without MSC treatment and OA joint treated with MSCs. The most significant pathways following functional enrichment analysis of the differentially abundant protein dataset were serine endopeptidase activity (*p* = 0.023), complement activation (classical pathway) (*p* = 0.023), and collagen containing extracellular matrix (*p* = 0.034). Due to the lack of an OA group without MSC treatment, findings cannot be directly correlated to only MSCs.

**Discussion:**

To date this is the first study to quantify the global extracellular vesicle proteome in synovial fluid following MSC treatment of osteoarthritis. Changes in the proteome of the synovial fluid-derived EVs following MSC injection suggest EVs may play a role in mediating the effect of cell therapy through altered joint homeostasis. This is an important step toward understanding the potential therapeutic mechanisms of MSC therapy, ultimately enabling the improvement of therapeutic efficacy.

## Introduction

Osteoarthritis (OA) is a common disease of the joint, and is the cause of up to 60% of all lameness cases in horses (1). It is a progressive degenerative musculoskeletal pathology of synovial joints and results from an imbalance of catabolic and anabolic processes affecting cartilage and bone remodeling (2). This is associated with pain, reduced mobility and impaired welfare. OA is a complex heterogeneous condition with multiple causative factors, including mechanical, genetic, metabolic and inflammatory pathway activation, with a non-functional joint as the shared endpoint (3). In the disease there is a loss of articular cartilage, reduced elastoviscosity of synovial fluid, thickening of subchondral bone, joint space narrowing and osteophyte formation (4). Currently, OA is predominantly diagnosed based on clinical signs and radiographic imaging, capturing changes that are typical of the later disease stages. Treatment is symptomatic, with no current cure available to rescue the joint environment. Biological cell-based therapies include autologous conditioned serum, platelet-rich plasma, and expanded or non-expanded mesenchymal stem cells (MSCs). These regenerative therapies have the potential to enhance repair of damaged tissues or organs (5). Mesenchymal stem cell (MSC) therapy most commonly uses cells derived from bone marrow or adipose tissue (6). They are highly proliferative, plastic-adherent, fibroblast-like cells that express CD44, CD90, CD105 and do not express MHC class II or CD45 (7). MSCs are able to modulate and down-regulate immune system activity, reducing inflammatory cytokines associated with acute inflammation (8). However, MSC preparations display substantial heterogeneity of cell types that varies between donors and between tissue sources, which has led to varying results in tissue regeneration studies (9). Selection of MSCs to generate homogenous MSC preparations thus has the potential to improve the therapeutic outcome of MSC therapies (10, 11). Integrin α10β1, a collagen-binding receptor originally discovered on chondrocytes (12) is also expressed by MSCs (13) and can distinguish MSCs from other cell types in MSC preparations. MSCs selected for the expression of integrin α10β1 (integrin α10-MSCs) have shown improved adhesion to chondral and subchondral lesions in explant studies, improved chondrogenic differentiation ability as well as improved secretion of the immunomodulatory factor prostaglandin E2 (PGE2) *in vitro*, compared to unselected cells (14). It has been demonstrated that to mitigate the progression of osteoarthritis in an equine talar impact model such MSCs reduce cartilage fibrillation and subchondral bone sclerosis (15). In addition, labeled integrin α10-MSCs have been shown to home to cartilage defects in rabbit model after intraarticular administration and to directly participate in the regeneration of the cartilage (16).

MSC secreted factors are believed to have a critical role in the therapeutic efficacy. Thus, there is a drive to investigate and potentially develop cell-free therapeutics including Extracellular Vesicles (EVs) that reflect the biophysical characteristic of “parent” cells (17). EVs are nanoparticles enveloped in a phospholipid bilayer membrane and are secreted by most mammalian cells. EVs facilitate intercellular communication through the paracrine action of protein, lipid and nucleic acid cargo. EV subtypes, namely microvesicles and exosomes have been demonstrated to act in a protective manner, but also pathologically, dependent on the “parent” cell phenotype and subsequent *in vivo* environment (18, 19). Previous evidence has shown that EVs may at least partially drive the therapeutic effect of MSC treatment, by increasing cellular proliferation and infiltration in exosome-mediated cartilage repair by promoting a regenerative immune phenotype, characterized by higher infiltration of CD163 + macrophages and a reduction in proinflammatory cytokines such as interleukin 1β (IL-1β) (20), tumor necrosis factor- α (TNF-α) and interleukin 6 (IL-6) (21).

In *in vitro* equine models of OA, it has been found that chondrocytes stimulated with inflammatory cytokines had a reduced inflammatory phenotype following treatment with MSC -EVs. MSC-EVs also have an anti-catabolic effect evidenced by decreased expression of matrix metalloproteinase 13 (MMP-13) (22, 23). These observations were conserved across different species. In mice it was demonstrated that MSC-EVs reinduced the expression of chondrocyte markers (type II collagen, aggrecan) but inhibited catabolic (MMP-13) and inflammatory (nitric oxide synthase; iNOS) markers. Thus the authors suggested that MSC-EVs act in a chondroprotective manner by inhibiting apoptosis and macrophage activation (24).

It is paramount to determine the mechanism of therapeutic action of MSCs and determine the contribution of secreted factors such as EVs in rescuing the OA phenotype, with the aim of producing a more targeted treatment. We demonstrate that intraarticular injection of MSCs affects protein cargo of synovial fluid EVs in an equine model of OA, decreasing the expression of proteins associated with pathways related to OA pathogenesis.

## Materials and methods

### Equine carpal osteochondral fragment model induction

The study was approved by the Danish Animal Experiments Inspectorate (#2020-15-0201-00602) as well as the Ethical Committee of the University of Copenhagen (project no 2020-016). OA was induced using a carpal osteochondral fragment-exercise model in a total of 6 Standardbred trotters (mares, 4 to 7 years of age). The model was previously described by McIlwraith et al. (1). OA was induced in the left carpus of all horses and the right carpus was sham operated on to serve as control. Horses were premedicated with a combination of romifidine 6 mg/100 kg (Sedivet^®^Vet, Boehringer Ingelheim Vetmedica, Missouri, United States), acepromazine 3 mg/100 kg (Plegicil Vet, Boehringer Ingelheim Vetmedica, Missouri, United States), atropine sulfate 0.5 mg/100 kg (Atropin, Aguettant Ltd, Bristol, United Kingdom), and butorphanol 3 mg/100 kg (Dolorex^®^, Ag Marin Pharmaceuticals, United States). Anesthesia was induced with ketamine 2.5 mg/kg (Ketador Vet, Richter Pharma AG, Oberosterreich, Austria) and midazolam 4 mg/100 kg (Midazolam “Accord”, Accord-UK Ltd, Barnstaple, United Kingdom). The horses were placed in dorsal recumbency and anesthesia maintained with isoflourane (Vetflurane, Virbac, Carros, France). Perioperatively the horses received flunixin meglumine 1.1 mg/kg (Finadyne, MSD Animal Health, New Jersey, United States), penicillin 22.000 IU/kg (Benzylpenicillin PanPharma, Brancaster Pharma, Surrey, United Kingdom), and gentamicin 6.6 mg/kg (Genta-Equine, Dechra Veterinary Products, Shrewsbury, United Kingdom). Arthroscopic portals were made in the right carpus and the carpal joint was inspected for abnormalities. In the left carpus an osteochondral “chip” fragment was made with an 8 mm curved osteotome in the dorsal margin of the third facet of the distal surface of the radial carpal bone at the level of the medial plica. The fragment remained attached to the plica. The debris was not flushed from the joint. The horses were stall rested for 14 days after surgery. From day 2 following surgery horses were walked by hand every day. Treadmill exercise was initiated on day 14 after surgery. The horses were exercised 5 days a week for 8 weeks through the following program: 2 min slow trot 16–19 km/h (4.4–5.3 m/s), 2 min fast trot 32 km/h (9 m/s), 2 min slow trot 16-19 km/h (4.4–5.3 m/s). From day 14 the horses were allowed free pasture exercise every day. At day 18 MSCs were injected intraarticularly into the left joint with the osteochondral fragment only. Horses were humanely euthanized at the end of the study and both joints underwent both gross and histological examination. This data is not included.

Aseptic arthrocentesis was conducted on osteochondral chip joints and sham control joints at specific time points: day 0, 18, 21, 28, 35, and 70, as shown in Table 1. The synovial fluid (SF) was centrifuged at 2,540 *g* at 4°C for 5 mins and then aliquoted into Eppendorf tubes, and snap frozen. After completion of collection, samples were transported on dry ice to the University of Liverpool, and stored at −80°C. For clarity, experimental groups were control (day 0-day 70), OA (day 0-day 18) and OA + MSCs (day 21–70) following MSC treatment of the OA group post sampling at day 18, as shown in Table 1.

**Table 1:** An overview of experimental groups and the longitudinal time points for SF collection.

		**Day**						
		***0 (prior to surgery)***	***18***		***21***	***28***	***35***	***70***
Group	*Sham Control*	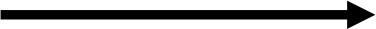		No treatment	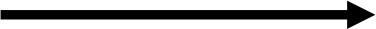			
	*OA*			MSC administration	OA + MSCs			
					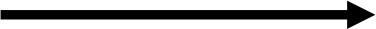			

OA + MSCs, Osteoarthritis and mesenchymal stem cells; OA, Osteoarthritis.

### Mesenchymal stem cell therapy

Equine MSCs were isolated from adipose tissue from a 7-year-old healthy gelding. Briefly, the adipose tissue was digested with collagenase, adipose cells were removed, and the stromal vascular fraction was isolated and expanded in culture. Cells were cultured to passage 3, and MSCs were then selected for high expression of integrin α10β1 (integrin α10-MSCs) by magnetic-activated cell sorting using a specific biotinylated integrin α10 monoclonal antibody (Xintela, Sweden) and anti-biotin microbeads (Miltenyi, North Rhine Westphalia, Germany). MSCs were subsequently washed in culture medium, reseeded and expanded for a further passage (passage 4), then frozen in 10% dimethyl sulfoxide (DMSO) cryopreservation medium (Cryostor, BioLife Solutions, Washington, United States) in liquid nitrogen until use. The frequency of MSCs expressing integrin α10β1 measured by flow cytometry, was 92.7% before cryopreservation.

On day 18 all six horses were treated with 20 × 10^6^ integrin α10-MSCs (4 ml, in 10% DMSO in cryopreservation medium) immediately after synovial sampling. Cryopreservation medium was not injected into the control joint. Day 18 was selected for MSC treatment to be administered to ensure an OA phenotype had developed within the joint as a response to surgical intervention. This was based on our previous study (25). The integrin α10-MSCs were thawed in a sterile water bath at 37°C, aspirated into a syringe through a 14G canula at a slow pace, and injected into the carpal joint of the OA-induced limb through a 20G canula over a minimum of 10 secs. The horses were stall rested for 2 days following treatment.

### Sample preparation

The 1 ml of SF per sample was spun in a benchtop centrifuge (Eppendorf non-refrigerated centrifuge 5420) at 241 *g* for 10 min. The supernatant was removed and treated with hyaluronidase (1 μg/ml) by incubation at 37°C for 1 h. SF samples were then spun at 1,000 *g* for 5 min, and the supernatant was collected.

### EV isolation—differential ultracentrifugation

Equine SF samples (200 μl) underwent differential ultracentrifugation (dUC) in order to isolate EVs. Samples were subjected to a 300 *g* spin for 10 min, 2,000 *g* spin for 10 min, 10,000 *g* spin for 30 min in a bench top centrifuge at room temperature. Samples were then transferred to Beckman Coulter thick wall polycarbonate 4 ml ultracentrifugation tubes, and centrifuged at 100,000 *g* for 70 min at 4°C (Optima XPN-80 Ultracentrifuge, Beckman Coulter, California, USA) in a 45ti fixed angle rotor, with the use of a 13 mm diameter Delrin adaptor. Supernatant was removed and sample pellets were resuspended in 50 μl of filtered phosphate buffered saline (PBS) (Gibco™ PBS, pH 7.4—Fisher Scientific, Massachusetts, USA).

### Nanoparticle tracking analysis

Nanoparticle tracking analysis (NTA) was used to quantify EV concentration and size in all samples, using a NanoSight NS300 (Malvern Panalytical, Malvern, UK). Samples were prepared as previously described (26).

### EV characterization

The ExoView platform (NanoView Biosciences, Malvern Hills Science Park, Malvern) was used to determine EV concentration, surface marker identification (CD9, CD81 and CD63) and to perform fluorescent microscopy and tetraspanin colocalisation analysis on selected samples. We had previously tested equine samples on both the human and murine chips and demonstrated the human chips were more compatible (data not shown). The ExoView analyzes EVs using visible light interference for size measurements and fluorescence for surface protein profiling. Samples were analyzed as previously described (25).

### EV protein extraction

EV pellets were suspended in 200 μl of urea lysis buffer (6M Urea (Sigma-Aldrich, Dorset, United Kingdom), 1M ammonium bicarbonate (Fluka Chemicals Ltd., Gillingham, UK) and 0.5% sodium deoxycholate (Sigma-Aldrich, Dorset, United Kingdom)). Samples were sonicated at 5 μm for 3 × 10 secs per sample, with 1-min rest on ice between each sonication round.

### SDS-PAGE and protein staining

Sodium dodecyl sulfate–polyacrylamide gel electrophoresis (SDS-PAGE) was used to separate proteins from EV protein extract. 7.5 ul of 2x Novex™ Tris-Glycine SDS Sample Buffer (ThermoFisher Scientific, Paisley, UK), supplemented with 8% of 2-Mercaptoethanol (Sigma-Aldrich, Dorset, UK), was added to 7.5 μl of sample SF-EV protein lysate. Samples were mixed and heated at 100°C for 10 min to denature proteins, then placed on ice. A NuPAGE™ 4 to 12%, Bis-Tris gel (ThermoFisher Scientific, Paisley, UK) was placed in the electrophoretic tank and the tank was filled with 1x NuPAGE^®^ MES Running Buffer (ThermoFisher Scientific, Paisley, UK) (diluted from the 20x stock in ultrapure water). Samples were loaded onto the gel alongside the Novex™ Sharp Pre-stained Protein Standard ladder (ThermoFisher Scientific, Paisley, UK). Gels were run at 100V until completion of electrophoresis and visualized using colloidal coomassie blue (Thermofisher Scientific, Paisley, UK) according to manufacturer's guidelines.

### In-solution digestion

95 μl of lysed and sonicated equine SF-EV were treated with 5 mM dithiothreitol (DTT) (Sigma-Aldrich, Dorset, UK) 100 mM at 60°C and 123 *g* for 30 mins. Iodoacetamide (Sigma-Aldrich, Dorset, UK) was then added to a final concentration of 20 mM and the samples were incubated at room temperature in the dark for 30 min. Following this 5 mM DTT was added to each sample, and incubated at room temperature for 15 min. 12 μl hydrophilic and hydrophobic magnetic carboxylate SpeedBeads (SP3 beads, total of 12 μl) (Cytiva, Massachusetts, United States) were added to each sample, followed by 120 μl ethanol (Sigma- Aldrich, Dorset, UK). Samples were then incubated at 24°C and 123 *g* for 1 h. The beads were separated from samples using a magnetic stand and were washed three times with 180 μl 80% ethanol. They were resuspended in 100 mM ammonium bicarbonate (Fluka Chemicals Ltd., Gillingham, UK4 μg). Trypsin/LysC (2.4 μg) (Promega) was added to each sample. Samples were placed in a sonicator bath and sonicated for 30 secs to disaggregate the beads before being incubated overnight at 37°C and 123 *g*. Beads were removed from the samples using the magnetic stand and the supernatants were acidified by the addition of 1 μl trifluoroacetic acid (Sigma- Aldrich, Dorset, UK). Samples were then desalted using an Agilent mRP-C18 column, dried in a SpeedVac and resuspended in 0.1% formic acid. The UV absorbance measured during desalting was used to normalize the loading for mass spectrometry analysis with a final volume of 5 μl being loaded on the nano-LC column.

### Data-dependent acquisition for generation of an equine SF EV spectral library

Equine SF was pooled using samples from the metacarpophalangeal joint from our equine musculoskeletal biobank (VREC561), and samples collected in this study from carpal joint of control group as well as OA, and OA + MSC group, resulting in a total of 11 ml SF. This was hyaluronidase treated (1 μg /ml) for 1 h at 37°C, as outlined in section 2.2. EVs were isolated using dUC, as outlined in section 2.4. The EV pellet was then reconstituted in 200 μl of urea lysis buffer. The samples were digested with trypsin/LysC for 3 h at 37°C, the concentration of urea was reduced to 1 M, and incubation was continued overnight at 37°C. Samples were fractionated on a PolySULFOETHYL, a strong cation exchange column, and 20 fractions were desalted, dried and resuspended in 0.1% formic acid. Aliquots were loaded onto an Eksigent nanoLC 415 (Sciex, Macclesfield, United Kingdom) equipped with a nanoAcquity UPLC Symmetry C18 trap column (Waters, Massachusetts, United States of America) and a bioZEN 2.6 μm Peptide XB-C18 (FS) nanocolumn (250 mm × 75 μm, Phenomenex, Macclesfield, United Kingdom). A gradient from 2–50% acetonitrile /0.1% formic acid (v/v) over 120 min at a flow rate of 300 nL/min was applied. Data-dependent acquisition was performed using nano liquid chromatography-tandem mass spectrometry on a Triple TOF 6600 (Sciex, Macclesfield, United Kingdom) in positive ion mode using 25 MS/MS per cycle (2.8 s cycle time), and the data were searched using ProteinPilot 5.0 (Sciex, Macclesfield, United Kingdom) and the Paragon algorithm (SCIEX) against the horse proteome (UniProt *Equus cabullus* reference proteome, 9,796, May 2021, 20,865 entries). Carbamidomethyl was set as a fixed modification of cysteine residues. The data were also searched against a reversed decoy database and proteins lying within a 1 or 5% global false discovery rate (FDR) were included in the library. Proteins were analyzed using FunRich.

### Data-independent acquisition proteomics

A data-independent proteomic approach was utilized in the form of Sequential Windowed Acquisition of all theoretical fragments (SWATH) (27). Aliquots of 5 μl containing equal quantities of peptides were made up to a volume of 5 μl with 0.1% formic acid and data were acquired using the same 2 h gradient as the library fractions. SWATH acquisitions were performed using 100 windows of variable effective isolation width to cover a precursor m/z range of 400-1500 and a product ion m/z range of 100-1650. Scan times were 50 ms for TOF-MS and 36 ms for each SWATH window, gave a total cycle time of 3.7 secs. Retention time alignment and peptide/protein quantification were performed by Data-Independent Acquisition by Neural Networks (DIA-NN) (28), using the same reference horse proteome as above to reannotate the library. A precursor FDR of 1%, with match between runs and unrelated runs was selected. The mass spectrometry proteomics data were deposited to the ProteomeXchange Consortium via PRIDE (29) (reference PXD035303).

### Statistical analysis

All statistical results were corrected for false discovery rate (FDR) using the Benjamini-Hochberg (BH) method unless stated otherwise. Results were considered significant at 5% FDR. Nanoparticle tracking analysis data was analyzed using non-parametric tests. A Kruskal-Wallis test was performed for concentration and size, followed by a Mann–Whitney U test per time point. Exoview data was analyzed using T-tests following parametric evaluation in GraphPad Prism 9.0. Statistical analysis of proteomics data was carried out using the R statistical programming environment (30), unless stated otherwise. The data was quality controlled; proteins with complete observations were normalized and log2 transformed for downstream analysis. Batch effect was detected and corrected via ComBat (31) prior to Principal Component Analysis (PCA) and further visualizations. The lmerTest implementation of lme4 (32) was used to fit linear mixed models (LMMs) to the log-transformed data for each protein to determine the effects of treatment, time, and treatment over time on horse joints. For the fitted models pairwise comparisons between the treatment and control were carried out at each time point using the emmeans package with the Kenward-Roger method (33). All graphical representations were undertaken using the package ggplot2 (34). Functional classification and enrichment analyses were performed using the clusterProfiler package (35). The proteins were annotated with GeneOntology (GO) terms using the UniProtKB ID Mapping tool. Over-representation analysis (ORA) was carried out for GO terms using the enricher function from the clusterProfiler package. The foreground was all the proteins that passed FDR, the background was all the processed proteins after missing values had been removed, i.e., all the proteins that were subjected to statistical analysis. Each term was required to have a minimum of three observed proteins annotated to it and an adjusted *p*-value < 0.05.

## Results

### Equine carpal osteochondral fragment model

The carpal osteochondral fragment-exercise model used in this experiment has been shown to result in an OA phenotype with respect to clinical parameters, (1, 36–38) and this model has been used determine the response to to integrin α10-MSC treatment, lameness and joint degradation were assessed but are not shown (Anderson et al. unpublished data).

### Nanoparticle tracking analysis to quantify EV size and concentration

**Figure 1:**
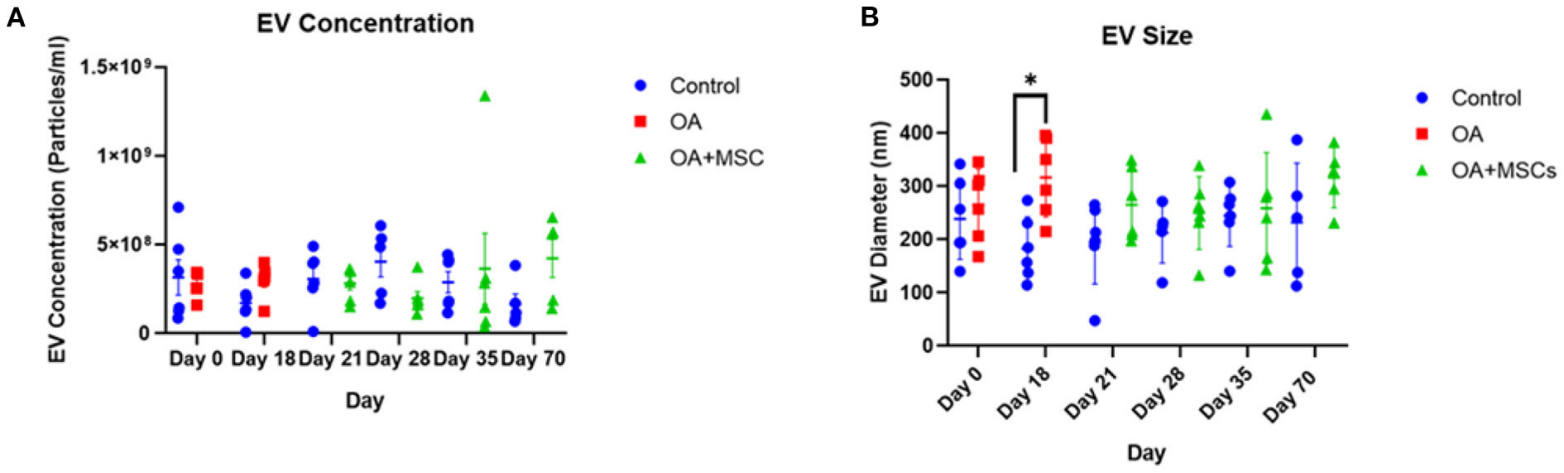
Size and concentration of synovial fluid-derived nanoparticles. Nanoparticle tracking analysis was undertaken using a Nanosight NS3000. All error bars are standard error of the mean (SEM). Statistical analysis undertaken in GraphPad Prism 9.0 using Kruskal Wallis tests with FDR correction and Mann Whitney tests within time points (*p* < 0.05, *; *p* < 0.01 **: *p* < 0.001, ***, *p* < 0.0001, ****). **(A)** EV concentration and **(B)** EV size.

Total particle concentration and size characterization was performed using NTA. NTA determined the average SF-EV sample concentration between control, OA and OA + MSCs across specific time points, specifically quantifying all nanoparticles within the sample (Figure 1A). No significant difference was observed in EV concentration between experimental groups, however a significant difference in EV size was found irrespective of time when comparing control and OA (*p* = 0.02) and control compared to OA + MSCs (*p* = 0.02). Specifically, EV size was significantly different between control and OA at day 18 before MSC treatment (*p* = 0.02) (Figure 1B). Results were suggestive of a heterogeneous population of nanoparticles.

### Exoview assay characterizes equine synovial fluid extracellular vesicles, including morphology, and surface tetraspanins

**Figure 2:**
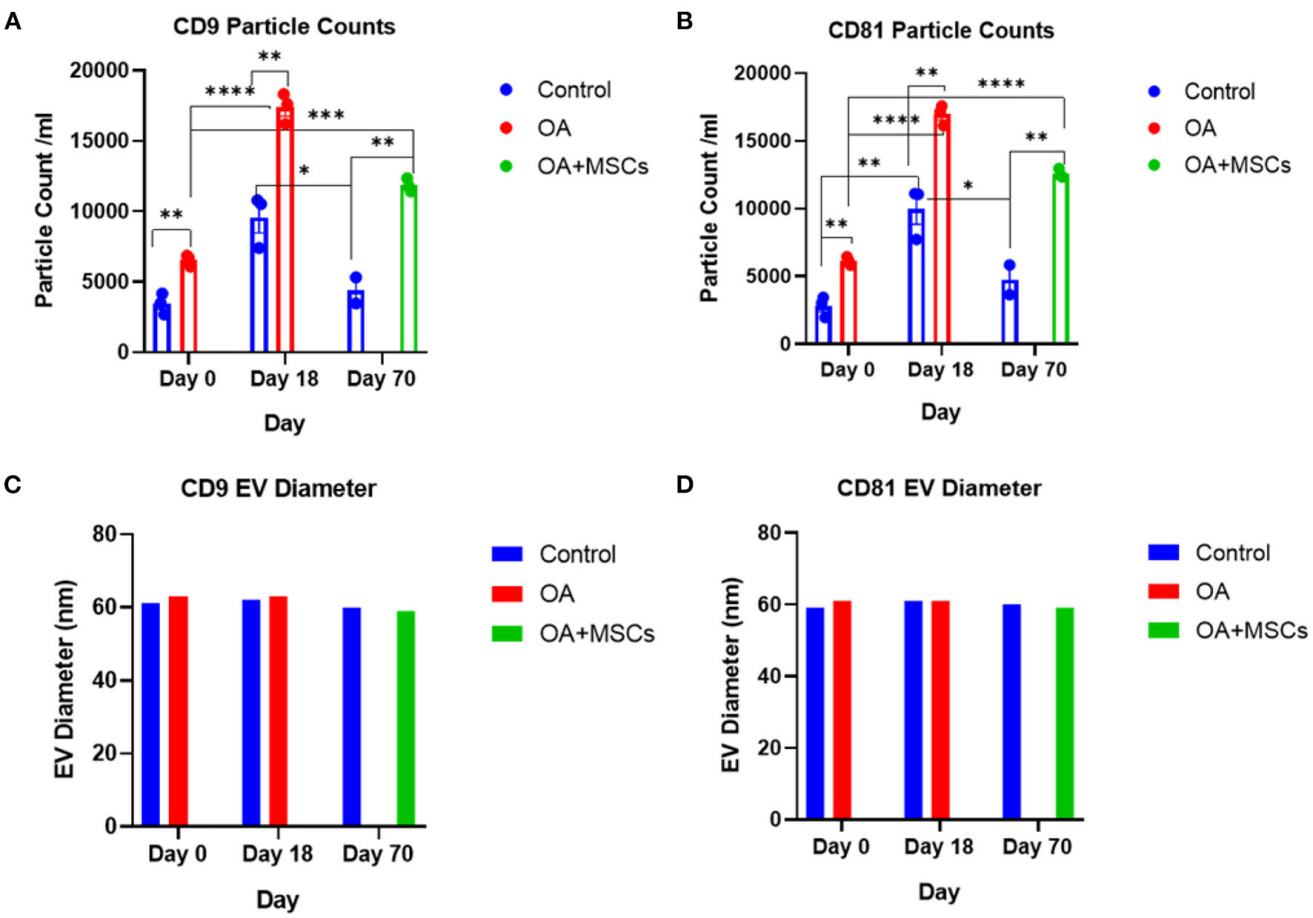
Sizing and enumeration of synovial fluid-derived EVs. All data was adjusted for dilution of the sample onto the chip. Shown is the average representing mean of three technical replicates that were run for each sample. Particle numbers were quantified by the number of particles in a defined area on the antibody capture spot. All bars are mean and standard error mean. **(A)** CD9 and **(B)** CD81-positive particles following probing with fluorescent tetraspanin antibodies. **(C)** Sizing of CD9 and **(D)** CD81 labeled EVs, normalized to MIgG control. Limit of detection was 50–200 nm. Statistical analysis undertaken in GraphPad Prism 9.0 using T-tests following parametric evaluation (*p* < 0.05, *; *p* < 0.01 **: *p* < 0.001, ***, *p* < 0.0001, ****).

**Figure 3:**
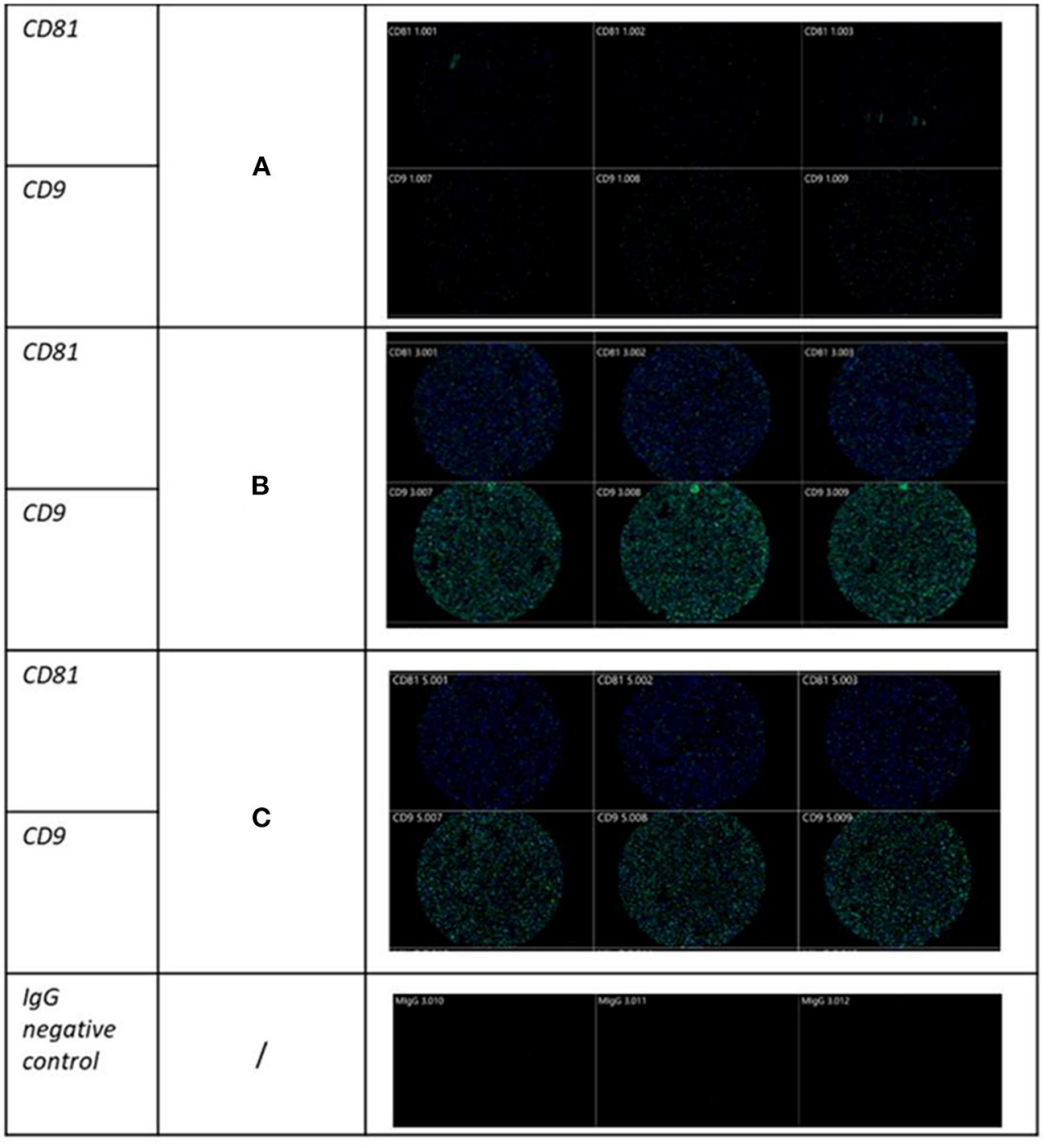
Visualization of SF-derived EVs from control, osteoarthritic (OA) and OA + MSCs joints using Exoview at selected time points. A fluorescent image of a representative spot is shown for each sample comparing **(A)** control, **(B)** OA, and **(C)** OA + MSCs with color denoting surface tetraspanin positive identification (blue-CD9, and green-CD81).

In addition to NTA, representative EV samples were characterized using the human exoview tetraspanin chip assay. This assay specializes in characterizing the exosome subpopulations of EVs. Control at day 0, OA and control at day 18 and OA + MSCs and control at day 70 were compared. OA and OA + MSCs groups had a significantly higher concentration of EVs when compared with controls. For CD9 expression, control had 4.63 × 10^3^ particles, OA had 21.91 × 10^3^ particles, and OA with MSCs had 15.97 × 10^3^ particles. Similarly, for CD81, control had 3.41 × 10^3^ particles, OA had 17.23 × 10^3^ particles and OA with MSCs had 12.48 × 10^3^ particles (Figure 2). CD63 was not reported due to low particle counts for this tetraspanin; this has been attributed to poor protein homology between equine and human CD63 tetraspanins. EVs were visualized between groups with fluorescent microscopy, highlighting tetraspanin expression and EV morphology (Figure 3).

#### Spectral library for equine synovial fluid extracellular vesicles

**Figure 4:**
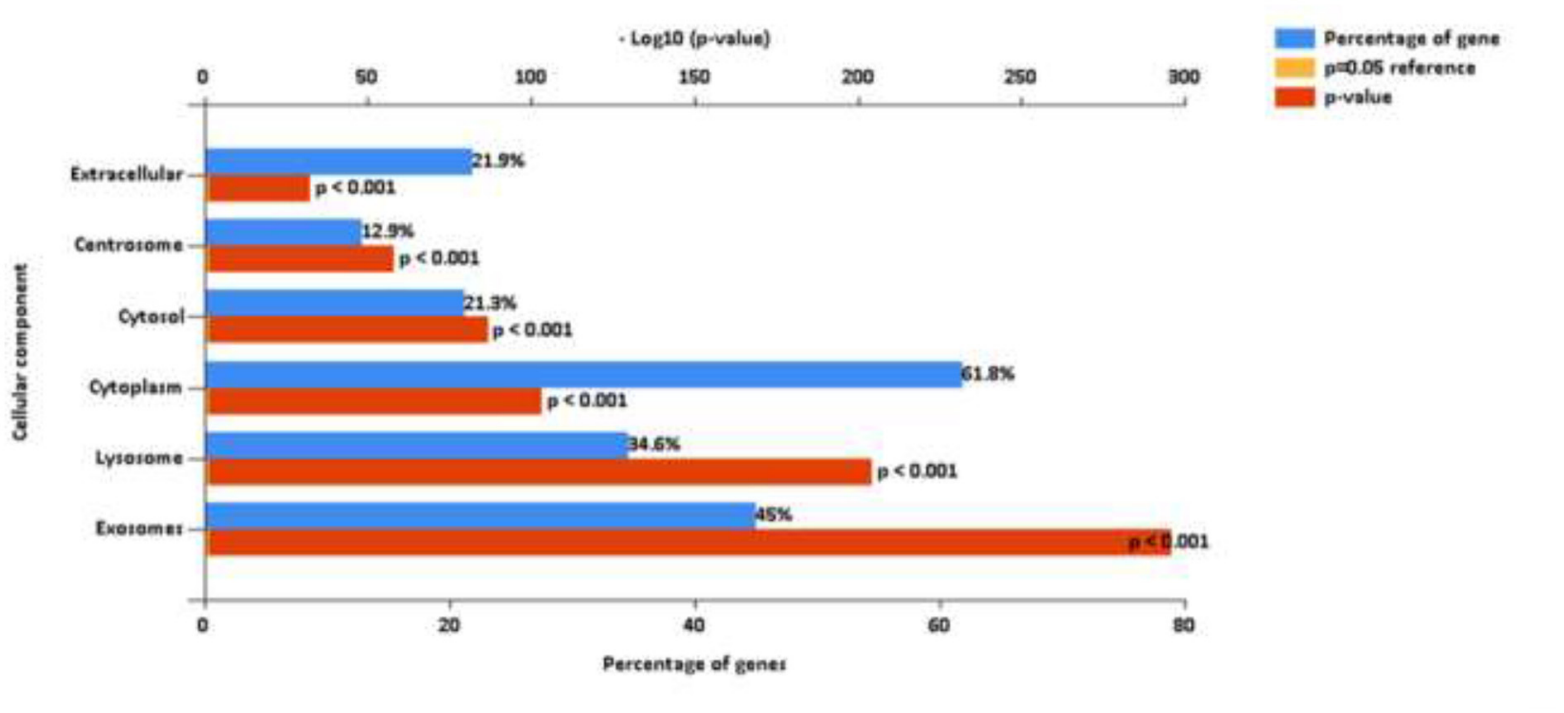
FunRich analysis output, after mapping 2,047 proteins to GO Cellular Component terms. These proteins were identified from a pooled sample of equine synovial fluid (11 ml) used to generate the SF-EV spectral library for this study. SF was sourced from healthy, OA and OA + MSC treated joints in order to encapsulate all potential proteins that may be present across all experimental groups.

Mass spectrometric analysis of the SF-EV pooled sample identified 2271 proteins, mapping at least one unique peptide. Of these proteins 2047 were identified and mapped to GO Cellular Component terms using FunRich. Proteins were attributed to various cellular components, including extracellular space and exosomes, both *p* ≤ 0.001, as shown in Figure 4. This library was then used to identify the proteins present within the individual samples.

### A multivariate approach identified a time-dependent difference between disease stages pre- and post-treatment

**Figure 5:**
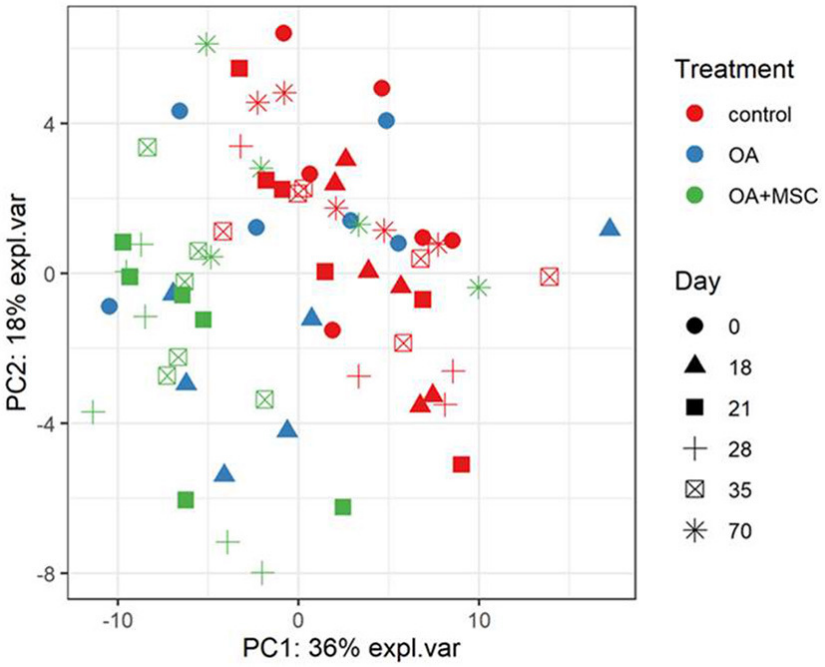
Multi-level PCA (mPCA). The first two principal components are plotted, accounting for ~54% of variance. Samples based on SWATH-MS. Each plotted point represents a horse, which are colored by their treatment and shaped by the day of the study.

A total of 442 proteins were identified across all samples submitted for SWATH-MS analysis. Multi-level PCA (mPCA) was carried out using the MixOmics R package principal component analysis (mPCA). PCA normally assumes the variables are not correlated. However, this study employed a repeat-measures design on the same horses. To account for the intraclass correlation between horses mPCA was employed. The first two principal components accounting for 54% of the variance were associated with the biological effect and demonstrated that the control and OA + MSC samples clustered by treatment (Figure 5). The later OA + MSC time points (day 70) appeared to cluster together with the control samples, reflecting a return to protein expression levels comparable to the healthy controls.

### Longitudinal differential expression highlights 6 key pathways related to disease progression and treatment

**Figure 6:**
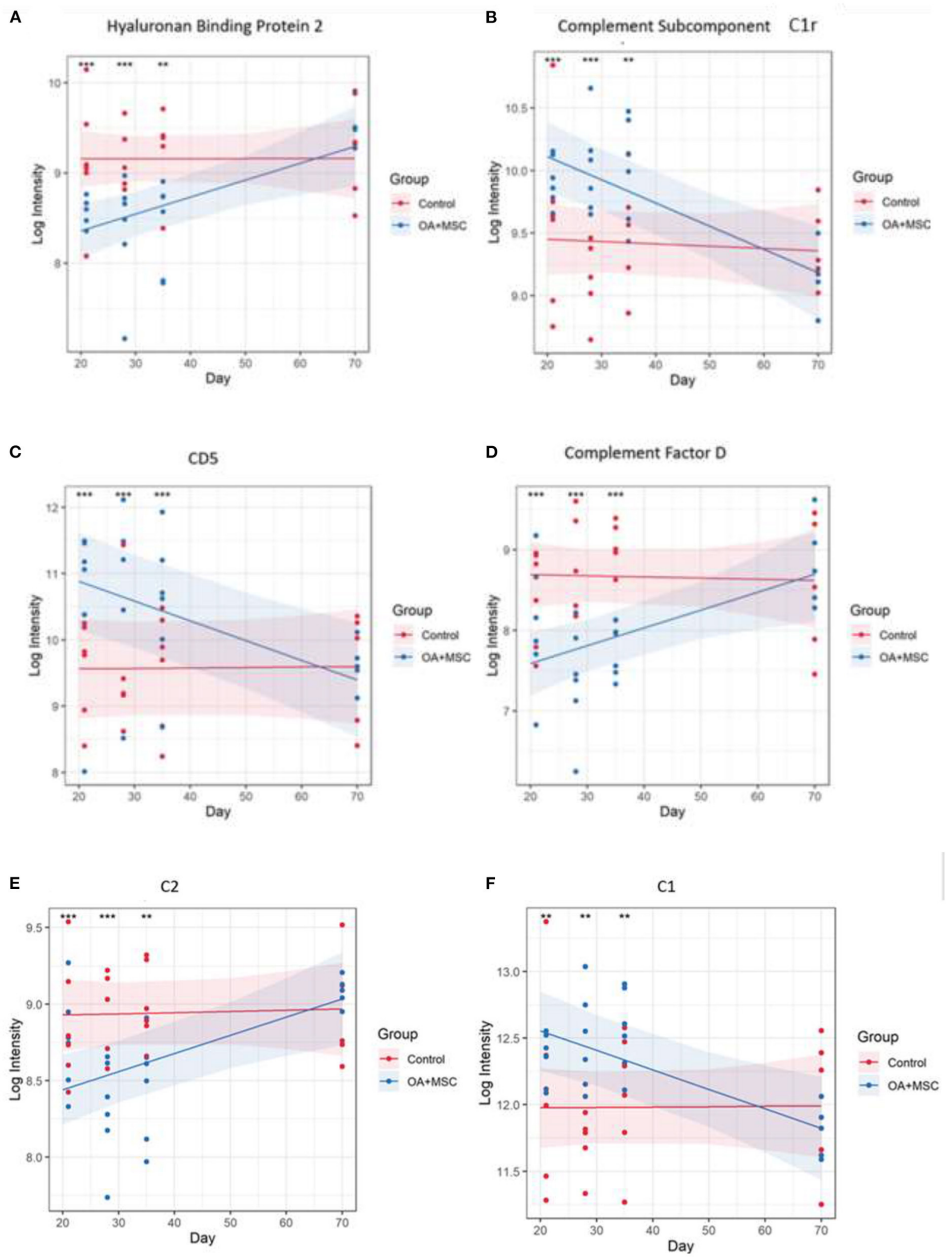
Protein expression changes in response to MSC treatment in OA joints vs control. **(A)** Hyaluronan binding protein 2, **(B)** Complement subcomponent C1r, **(C)** CD5, **(D)** Complement factor D, **(E)** C2, and **(F)** C1. The models were fitted using the lmerTest implementation of lme4. 48 proteins had a significant group, time, or group:time effect after FDR correction. For each protein with a significant effect the model was plotted using the effects and ggplot2 packages. The fitted model is shown as a line for the OA + MSC (blue) and control (red). The 95% confidence intervals for each group are shown as a shaded area. The raw data is included as points. At each time point pairwise comparisons were carried out between the treatment and control using the emmeans package. Significance thresholds were as follows: (*p* < 0.05, *; *p* < 0.01 **: *p* < 0.001, ***, *p* < 0.0001, ****).

Differentially expressed (DE) proteins were identified with the application of a linear mixed model with regressed factors comparing protein expression between experimental groups across time. Statistical significance was attributed to any protein with an FDR corrected *p*-value (BH) of p≤ 0.05 meeting a minimal 95% confidence interval. Of the 442 proteins identified, 48 were present at significantly different levels (p≤ 0.05) between control and OA + MSCs regardless of time. Interestingly, there were no proteins DE after FDR correction in SF-EVs between control and OA at baseline and day 18 after OA induction, or between day 18 sham control and OA. The 10 proteins with the most statistically significant altered levels are shown in Table 2, including the time points at which pairwise comparisons show significant differences between control and OA + MSCs, and all 48 can be found in Supplementary Table 1. Figure 6 shows proteins attributed to the serine endopeptidase (*p* = 0.02) and complement pathways (*p* = 0.03), included hyaluronan binding protein 2 (*p* = 0.04) (Figure 6A), complement subcomponent C1r (*p* = 0.03) (Figure 6B), CD5 (*p* = 0.01) (Figure 6C), complement factor D (*p* = 0.03) (Figure 6D), C2 (*p* = 0.04) (Figure 6E), and C1 (*p* = 0.04) (Figure 6F). Other proteins attributed to the serine endopeptidase pathway include haptoglobin (*p* = 0.03), HtrA1 serine peptidase (*p* = 0.03) and complement factor B (*p* = 0.04). Proteins mapped to the collagen containing extracellular matrix (*p* = 0.02) included cartilage oligomeric matrix protein (*p* = 0.001), microfibril associated protein 4 (*p* = 0.001), thrombospondin 4 (*p* = 0.003), retinoic acid receptor responder protein 2 (*p* = 0.03), periostin (*p* = 0.03), EGF containing fibulin extracellular matrix protein 1 (*p* = 0.03), and cartilage intermediate layer protein (*p* = 0.04) (Supplementary Figure 1). The third most significant pathway was identified as complement activation classical pathway, with the following proteins attributed to it; two uncharacterized proteins, C9 (*p* = 0.01), C8A (*p* = 0.02), C1r (*p* = 0.03), C7 (*p* = 0.03), C2 (*p* = 0.04), and C1 (*p* = 0.04) (Supplementary Figure 1). Across all figures (Figures 6A, 4F) protein expression was significantly different at day 21, 28 and 35 when compared to control. All protein expressions returned to baseline control by day 70.

**Table 2:** The top 10 differentially expressed (*p* < 0.05) proteins following the application of the linear mixed model, accounting for treatment and time point.

**Protein**	**Accession number**	***P*-Value (FDR adjusted)**	**Time point** **(Day)**	**Expression direction in OA + MSC group compared to control**
Fibrinogen beta chain	F6PH38	0.0001	21,28,35	Increase
Fibrinogen gamma chain	A0A5F5PPB8	0.0001	21,28,35	Increase
Joining chain of multimeric IgA and IgM	A0A3Q2HW24	0.0003	21,28,35	Increase
Dynein heavy chain domain 1	A0A3Q2HE28	0.0005	21,28,35	Increase
Fibrinogen alpha chain	A0A3Q2HTG2	0.0005	21,28,35	Increase
Gelsolin (Actin-depolymerizing factor, ADF) (Brevin)	Q28372	0.0006	21,28,35	Decrease
Cartilage oligomeric matrix protein	A0A3Q2HRL2	0.001	21,28,35	Decrease
Microfibril associated protein 4	A0A3Q2HNH0	0.001	21,28,35	Increase
Glutathione peroxidase	A0A5F5PST7	0.003	21,28,35	Decrease
Insulin like growth factor binding protein 6	F7DEB1	0.003	21,28,35	Decrease

OA + MSCs, Osteoarthritis and mesenchymal stem cells; IgA, Immunoglobulin A; IgM, Immunoglobulin M.

Treatment and time were included as main effects, along with a treatment-by-time interaction term. Time point denoting significant pairwise comparisons between control and OA + MSCs is also shown.

**Figure 7:**
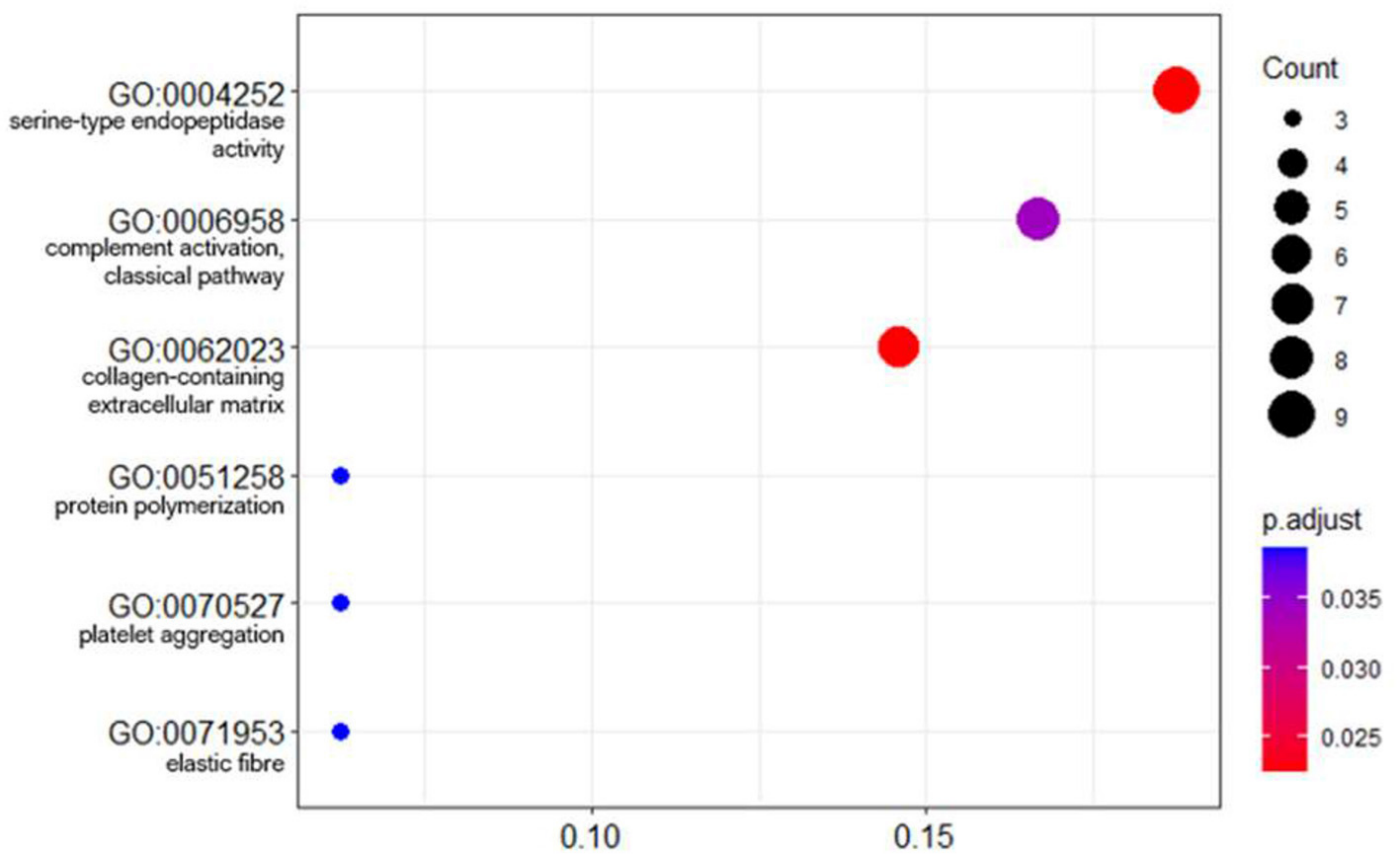
Dot plot of GO term enrichment analysis of differentially abundant proteins. The size of the dots indicates the number of proteins that mapped to that term. The x-axis is the protein ratio (number of proteins that map to the term divided by the total number of significant proteins). The dots are shaded by adjusted *p*-values (BH method).

The lists of significant proteins were used to calculate functional enrichment with the aim of identifying relevant biological processes representative of the signature. Using an overrepresentation analysis (ORA) approach on gene ontology (GO) annotations yielded six enriched pathways: serine endopeptidase activity (*p* = 0.023), complement activation (classical pathway) (*p* = 0.023), collagen containing extracellular matrix (*p* = 0.034), protein polymerization (*p* = 0.039), platelet aggregation (*p* = 0.039), elastic fiber (*p* = 0.039) (Figure 7).

## Discussion

This study aimed to determine the EV protein cargo following integrin α10β1-selected mesenchymal stem cell (integrin α10-MSC) treatment in an experimental model of equine OA. To our knowledge this is the first study of its kind to quantify the global EV proteome *in vivo* after MSC treatment.

The osteochondral fragment model used in this study has been shown to produce reliable post-traumatic OA in the middle carpal joint of horses (1, 36–38). The mitigating effect of MSC treatment on the development of post-traumatic OA has been shown in a number of equine studies, although in different models, such as an osteochondral fragment model of the fetlock (39, 40) a blunt impact model of the tarsus (15) and injection of the irritant amphotericin B (41). In these studies, the MSC-treated horses developed significantly less severe OA over time compared to the untreated control horses in terms of decreased synovial effusion and a higher SF viscosity and glycosaminoglycan content, decreasing lameness over time, less severe macroscopic cartilage erosions, less radiographic signs of OA, and less histologic cartilage fibrillation and subchondral sclerosis. It should be highlighted that in the carpal osteochondral fragment model used by Frisbie et al. (37) a significant change was observed in response to surgical induction of OA. Both adipose derived MSCs and bone marrow derived MSCs were used as treatment. Despite improvement being seen with treatment compared with placebo with respect to clinical parameters, in agreement with previous studies, no significant difference was determined.

The model induced a significant change in all but two parameters, no significant treatment effects were demonstrated, with the exception of improvement in synovial fluid effusion PGE2 levels with bone marrow-derived mesenchymal stem cells when compared to placebo. A greater improvement was seen with bone marrow-derived mesenchymal stem cells when compared to adipose-derived stromal vascular fraction and placebo treatment.

In our study, we identified a global change in the EV proteome, and identified a possible mechanism of MSC therapy. A time-dependent change in the EV protein cargo was also observed, suggestive of a time associated therapeutic effect, which is in line with previous reports of the effects of MSC-treatment.

We used dUC to isolate EVs following hyaluronidase treatment of SF. Hyaluronidase was used in order to break down hyaluronic acid as its presence in SF increases viscosity making the biofluid difficult to handle. This pretreatment is known to increase EV yield (35). In addition, it has also been suggested that SF-derived EVs should be sedimented at a speed of at least 100,000 *g* for optimal EV recovery, hence the decision to use this step within our isolation protocol (35). There are many EV isolation protocols with no standardized protocol agreed upon. Each isolation method results in different sample concentrations, purity, and EV profiles/ heterogeneity.

EVs were isolated from SF obtained from OA joints that were treated with MSCs and from untreated control joints. The EVs isolated were a heterogenous population that may have been derived from cells found in the intraarticular environment, such as synoviocytes and chondrocytes, and possibly from the MSCs injected into the joint. Nanoparticle tracking analysis conducted on all samples across all time points found no changes in EV concentration with time. It should be noted that NTA quantifies all nanoparticles within a sample and includes lipoproteins, proteins, viruses, nanovectors and drug delivery systems (42). This accounts for the difference in EV concentration between our NTA analysis and exoview analysis. We had limited resources to enable Exoview of all samples, and so used the platform for a subset of samples. Exoview analysis specifically focuses on the exosomal population of EVs by using antibodies for surface tetraspanins such as CD9, CD81 and CD63. In this study and our previous study, we were able to show species cross reactivity with the CD9 and CD81 tetraspanins, but not CD63 (25). Exoview analysis demonstrated an increase in the number of exosomes after OA-induction surgery prior to MSC injection. However, this may be due to immune cell infiltration within the first 18 days contributing to the difference. This is in contrast to a human study by Mustonen et al. which identified no change in EV concentration between SF-EVs from healthy and human late stage OA patients (43). Of course, differences could be due to the stage of disease post-traumatic model used in our model (early) versus the end-stage nature of the human study. Our results tentatively suggest that the OA-induction surgery actively increases the number of exosomes as a result of the acute trauma. This could be due to the access of subchondral bone to the SF environment following production of an osteochondral fragment or from tissues within the joint as a response to the formation of the fragment or both. However, our experimental design does not enable us to decipher this. A greater number of CD9 + and CD81 + EVs were identified across all experimental groups using the exoview assay. CD9 + exosomes have been postulated to be a target for inflammatory regulation in specific pathologies (31). In addition, the increase count observed in OA + MSC groups compared with control could be attributed to its presence on hematopoietic cells, and the role of CD9 in regulating hematopoietic stem cells differentiation (44). With respect to CD81, there is limited literature available postulating the role of specifically CD81 + EVs. However, the tetraspanin CD81 is involved in providing a scaffold enabling the recruitment of complementary proteins, enabling the function of many cellular processes. CD81 expression has been strongly associated with cancerous pathologies, and has been shown to promote tumor growth and metastasis in human melanoma, while its knockdown in osteosarcoma models has reduced tumor progression (45). Across all experimental groups the sham control remained significantly lower than the experimental group at all time points, suggestive of a minimal effect from the arthroscopy in the sham control groups. In addition, highlighting that there was a limited systemic effect from the surgical induction of OA.

In this study, SF samples following OA induction and then following the addition of MSCs to the joint were available up to day 70 of treatment post-induction. Unfortunately, SF samples from OA joints without MSC treatment were not available for this study. This makes it difficult to be definitive about the source of the EVs in the SF following addition of MSCs. We believe these will be a combination of tissue-derived and MSC-derived EVs, resulting from MSC-to-cell interactions, cell-to-MSC interactions, and cell-to-cell interactions. Allogeneic MSCs have been traced in the joint of an ovine OA model up to 14 weeks after injection (46) and up to 12 weeks after injection in an OA model in rats (47). Therefore, it is possible that MSCs were present in the joint at study termination 52 days (7½ weeks) after MSC-injection.

Post intraarticular injection of MSCs an increase in EV COMP expression was observed returning to baseline control by day 70. COMP is a key protein present in cartilage extracellular matrix and is a target of degradation in early OA (48). In addition, proteins such as gelsolin, which had increased expression during the OA + MSC group, has previously been attributed to chondrocyte migration. It could be postulated that proteins highly associated with the joint may be sourced from EVs secreted by joint cells, raising the question of how EVs interact in their *in vivo* environment in response to stimuli.

The most significant GO term associated with DE EV-proteins was serine endopeptidase activity following MSC treatment. Serine type endopeptidases, or serine proteinases have been attributed to proteolytic cartilage destruction. In addition, serine proteinases perform vital functions such as cytokine regulation and receptor activation (49). Degradomic studies have demonstrated that an increase in proteases activity in OA, such as in HtrA1 was responsible for cartilage proteolysis (50). HtrA1 decreased across time following MSC injection. In a murine model the genetic removal of HtrA1 delayed the degradation of articular or condylar cartilage in mice (51). Moreover, a previous study profiling the synovial fluid-derived EV proteome cargo in OA patients of both sexes identified sex-specific differences in cargo, with enriched pathways including proteins involved in endopeptidase activity, specifically in women (52). This is of note as all horses included in this study were mares. With respect to serine endopeptidase activity in MSCs it has previously been reported that interactions between BM-MSCs and natural killer cells are fundamental to improving MSC therapeutic efficacy. It has been stated that serpin B9 has a cytoprotective function in MSCs (53). In other diseases such as colorectal cancer, the use of MSCs identified serpins as having immunomodulatory effects, acting on immune cells in order to induce a wound healing phenotype, as well as angiogenesis and epithelial to mesenchymal transition (54). Therefore, we hypothesize that the change in SF-EV proteome post MSC injection has the capacity to affect the serine endopeptidase pathway that is known for its detrimental effect on cartilage degradation and further animal studies are required to elucidate this.

A further altered GO term included collagen containing extracellular matrix, often linked with joint homeostasis. Exosomes from embryonic MSCs were found to balance synthesis and degradation of the cartilage extracellular matrix in an *in vitro* murine model (55). In our study, we observe a significant change in the expression of proteins associated with cartilage structure, such as COMP, hyaluronan binding protein 2, cartilage intermediate layer protein, chondroadherin and gelsolin. These proteins return to baseline control level by the end of our study. This suggests a restorative effect and return toward a healthy cartilage phenotype. It may be that such increased expression is more likely to be attributed to EVs secreted by native tissues than MSCs, which contribute to collagen extracellular matrix homeostatic function which could be beneficial in OA treatment. In addition, MSCs could be upstream regulators of these effects from native tissues. Hence it is possible that EVs from the native environment and MSCs are acting in concert with additional secreted factors to result in a therapeutic benefit.

In this study, the level of complement proteins in SF EVs decreased with time and returned to baseline in horses treated with MSCs, potentially affecting disease progression. The complement cascade was also a significantly enriched pathway. This pathway is activated in the early stages of OA, with C3a and C5a attributed to OA progression (56). In addition, it has been linked to extracellular cartilage matrix degradation, chondrocyte and synoviocyte inflammatory responses, cell lysis, synovitis, disbalanced bone remodeling, and osteophyte formation (57). Several complement components have previously been identified as upregulated in OA SF. It was reported that C3a and C5a promoted chemotaxis of neutrophils and monocytes, and increased leukotriene synthesis (58). In our study multiple complement factors were identified, including C7, C8, C9 and C2 (C3/C5 convertase) when comparing control to OA + MSCs across time. As such emphasizing that MSC therapy may act through the complement pathway in alleviating OA symptoms, or initiate a change in intrinsic tissues, altering the subsequent SF-EV proteome, reducing the effect of such pathway.

There are a number of limitations to our study. The duration of the study only enabled the quantification of the effect of MSCs *in vivo* on the global EV proteome in the short term. MSC viability following treatment could not be quantified. Thus, there is an inability to determine the number of EVs contributing to the SF-EV proteome derived from injected MSCs and those from joint tissues themselves.

The purpose of this study was to characterize EVs in the SF after MSC administration in equine joints with OA. The specific role of EVs secreted by the MSCs only still need to be elucidated through comparisons to untreated OA joints. One way to study MSC-derived EVs specifically could be to identify MSC-derived EVs based on surface markers.

The severity of OA phenotype is also a limitation, as the model has been shown to produce a post-traumatic OA phenotype with respect to clinical parameters, but molecularly there were no significant proteins identified between control and OA. Previous studies using the same OA model have shown an increase in the SF concentration of PGE2 and glycosaminoglycans in the OA joints shortly after OA induction (within the first two weeks) and an increase in the matrix degradation products CPII, CS846 and C1,2C only toward the end of the 70-day study period (59). Prior to FDR correction ANOVA identified 39 DE proteins with respect to group (control and OA), including: Spondin-1 (*p* = 0.005), HtrA1 serine endopeptidase (*p* = 0.02), and serotransferrin (*p* = 0.01), all of which have been previously implicated in OA pathogenesis (60–62). Thus, whilst we cannot be certain there appears to be an effect on the EV proteome following OA induction that would require further exploration with additional animal studies.

It needs to be determined if the MSC-EVs were acting in a causative or reactionary manner to the *in vivo* environment. There is a significant degree of variability with respect to MSC properties dependent on the donor or tissue source. Our study used integrin α10β1-selected adipose-derived MSCs and different results may have been achieved with an alternative source of MSCs or different MSC preparation methods. A study by Roelefs et al. found that synovium-derived adult GDF5-lineage MSCs had a significant role in response to joint injury (63). Thus, it is likely that the EV cargo will be MSC source dependent, potentially for the MSC-derived EVs as well as the joint tissue-EV response to MSCs. Another study by Broeckx et al. have shown reduced signs of OA following injection of chondrogenically induced peripheral blood-derived MSCs (64) in horses with induced OA. Barrachina et al. showed that proinflammatory primed MSCs have improved immunomodulatory abilities in the equine joint compared to naïve MSCs (41). A recent study showed that integrin α10-MSCs were able to home to a cartilage defect in rabbits and to directly participate in cartilage regeneration through chondrogenic differentiation *in vivo* (16), which has not previously been demonstrated with other MSC preparations. Therefore, we acknowledge that results of this study could have been different if another cell type was used.

**Figure 8:**
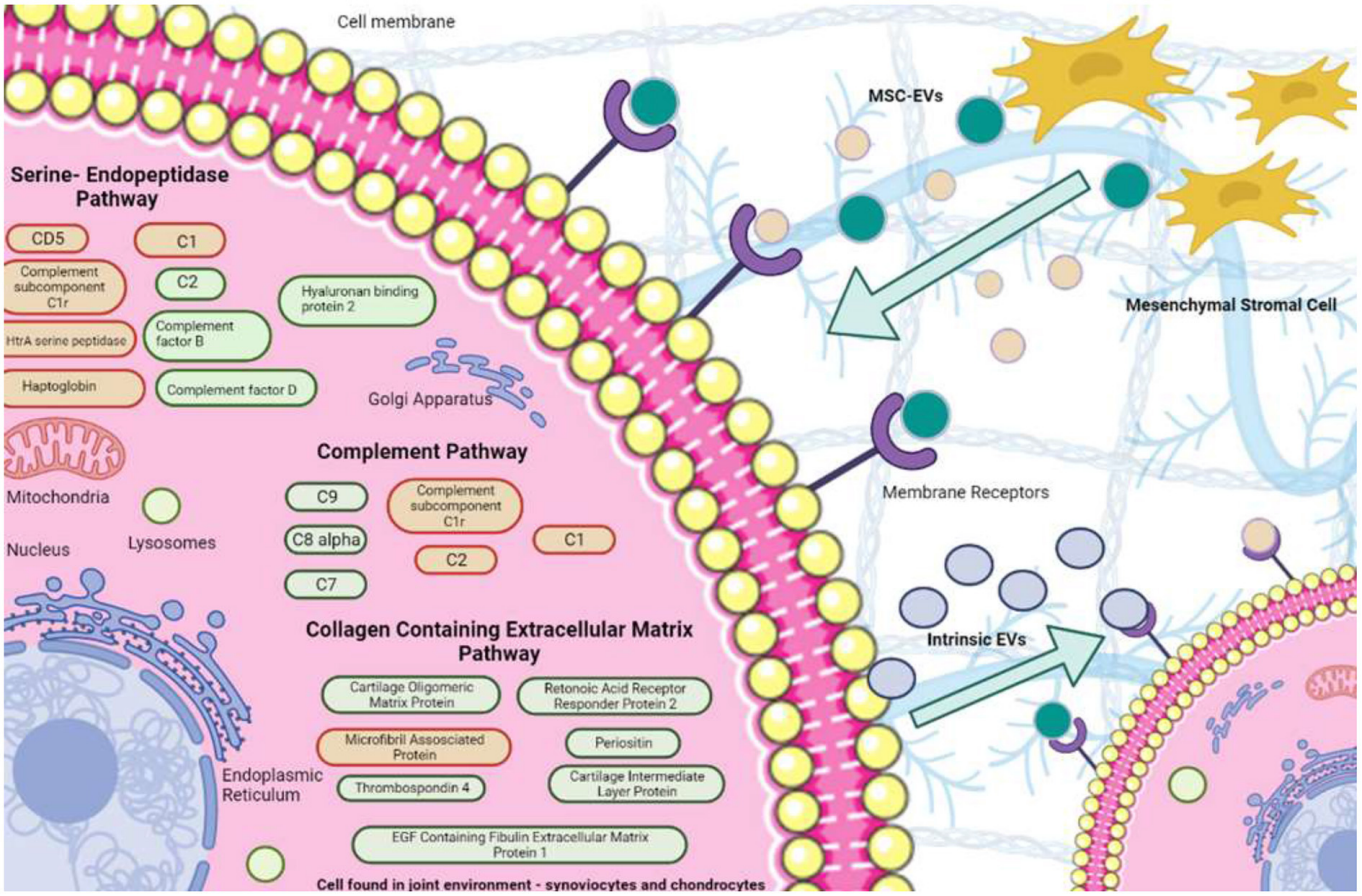
Potential mechanisms of action of SF-EVs following MSC treatment. EVs were sourced from both MSCs and the intraarticular environment. We hypothesized that MSC-EVs affect the intraarticular cells through their differential protein cargo, resulting in altered EV secretion from intrinsic cells. DE proteins attributed to given pathways are red (decreased expression to meet baseline or surpass it by day 70) or green (increased expression to reach baseline at day 70).

With the caveat that we did not have a group of horses with OA but no MSC treatment and that we were unable to confirm the MSC survival time, we have postulated a potential mechanism of action of MSCs in our model. We hypothesize that after the introduction of MSCs into the joint, MSC-EVs deliver proteinous cargo into recipient cells found within the intra-articular environment, while also promoting intrinsic cellular changes altering the cargo of EVs secreted from native cells. We suggest they act partially through effects on the serine endopeptidase pathway, subsequently reducing its activity and OA pathogenic effect. In addition, altered EV proteins are implicated in the complement system and collagen containing extracellular matrix pathway, as shown in Figure 8. These altered pathways may provide potential targets for therapeutic intervention and require further exploration in the context of MSC therapy and the use of MSC-EVs in OA. As such additional animal studies to assess if changes identified are due to the addition of MSCs to a joint in which OA has been induced or due to response of the joint to OA induction.

## Conclusion

We characterized for the first time using an unbiased approach the SF-EV protein cargo in a model of OA after MSC administration. Changes in the proteome of the synovial fluid-derived EVs following allogeneic integrin α10-MSC administration are suggestive of EVs playing a role in mediating the effect of cell therapy. A time-dependent change in potential therapeutic efficacy of the injected MSCs was also observed. Potential targets were identified that warrant further investigation in order to determine their significance in pathophysiology and management of equine OA.

## Data availability statement

The datasets presented in this study can be found in online repositories. The names of the repository/repositories and accession number(s) can be found below: The mass spectrometry proteomics data were deposited to the ProteomeXchange Consortium via PRIDE (21) (Reference: PXD035303).

## Ethics statement

The animal study was reviewed and approved by Danish Animal Experiments Inspectorate (#2020-15-0201-00602) and the Ethical Committee of the University of Copenhagen (Project No: 2020-016).

## Author contributions

MP, VJ, and SJ: conceptualization. EC, MP, SJ, CA, LB, EJ, ECG, RJ, and AT: methodology. EC, EJ, ECG, and RJ: formal analysis and visualization. EC, EJ, and RJ: investigation and data curation. EC: writing—original draft preparation. EC, MP, SJ, CA, LB, EJ, ECG, RJ, AT, CL, KU, and EL-Å: writing—review and editing. All authors have read and agreed to the published version of the manuscript.

## Funding

EC is a self-funded Ph.D. student. MP is funded through a Wellcome Trust Intermediate Clinical Fellowship (107471/Z/15/Z). This work was supported by the Horserace Betting Levy Board in conjunction with the racing foundation (SPrj048). Our work is also supported by the Medical Research Council (MRC), Xintela AB, and Versus Arthritis as part of the MRC Versus Arthritis Center for Integrated research into Musculoskeletal Aging (CIMA). This work was funded by the Horserace Betting Levy Board (HBLB), project code SPrj048.

## Conflict of interest

Authors EL-Å and KU were employed by Xintela AB and have shares in the company. The remaining authors declare that the research was conducted in the absence of any commercial or financial relationships that could be construed as a potential conflict of interest.

## Publisher's note

All claims expressed in this article are solely those of the authors and do not necessarily represent those of their affiliated organizations, or those of the publisher, the editors and the reviewers. Any product that may be evaluated in this article, or claim that may be made by its manufacturer, is not guaranteed or endorsed by the publisher.
