# Supplementary material for: Temporal extracellular vesicle protein changes following intraarticular treatment with integrin α10β1-selected mesenchymal stem cells in equine osteoarthritis
Source: Front Vet Sci. 2022 Nov 24;9:1057667. doi: 10.3389/fvets.2022.1057667 (PMC9730043; doi:10.3389/fvets.2022.1057667)
Supplement: Supplementary file 1 [file Data_Sheet_1.pdf]

Supplementary Tables and Figures

Table 1. A table showing all 48 significant proteins, with accompanying accession number, FDR corrected P-Value, and direction of expression from MSC injection into the joint, to the end of the 70 day study. All proteins returning to baseline control after 70 days.

**Protein**	**Accession number**	**P-Value (FDR adjusted)**	**Expression direction in OA+MSC group compared to control**
Fibrinogen beta chain	F6PH38	0.000124457	Increase
Fibrinogen gamma chain	A0A5F5PPB8	0.00012875	Increase
Joining chain of multimeric IgA and IgM	A0A3Q2HW24	0.000256117	Increase
Dynein heavy chain domain 1	A0A3Q2HE28	0.000467924	Increase
Fibrinogen alpha chain	A0A3Q2HTG2	0.000527871	Increase
Gelsolin (Actin-depolymerizing factor, ADF) (Brevin)	Q28372	0.000558416	Decrease
Cartilage oligomeric matrix protein	A0A3Q2HRL2	0.001269811	Decrease
Microfibril associated protein 4	A0A3Q2HNH0	0.001467511	Increase
Glutathione peroxidase	A0A5F5PST7	0.002535091	Decrease
Insulin like growth factor binding protein 6	F7DEB1	0.003249662	Decrease
Uncharacterized protein	A0A3Q2H908	0.003249662	Increase
Thrombospondin 4	F7E0P3	0.003398605	Decrease
Annexin	F7DE06	0.00767001	Increase
Chondroadherin	F6WD70	0.00767001	Decrease
Ectonucleotide pyrophosphatase/phosphodiesterase 2	A0A3Q2HYR4	0.009877629	Decrease
Sulfhydryl oxidase, EC 1.8.3.2	F6WR95	0.010834212	Decrease
CD5 molecule like	A0A3Q2HC10	0.011838064	Increase
Complement component C9	P48770	0.017797177	Decrease
Complement C8 alpha chain	A0A5F5PQS3	0.029587066	Decrease
Complement subcomponent C1r, EC 3.4.21.41	F6Z5L1	0.030381695	Increase
Histone H3	F6UU57	0.031141324	Increase
Fibulin-1	A0A3Q2GXX5	0.031158385	Decrease

72 kDa type IV collagenase, EC 3.4.24.24 (72 kDa gelatinase) (Matrix metalloproteinase-2)	A0A3Q2H348	0.032777103	Decrease
Retinoic acid receptor responder protein 2 (Chemerin)	F7C5F1	0.032777103	Decrease
HtrA serine peptidase 1	A0A3Q2KX04	0.032777103	Increase
Rho-associated protein kinase 2, EC 2.7.11.1 (Rho-associated, coiled-coil-containing protein kinase 2) (Rho-associated, coiled-coil-containing protein kinase II) (p164 ROCK-2)	F6QSI7	0.032777103	Decrease
Serpin family F member 1	F7BKE1	0.032777103	Decrease
Periostin	F6Y0G5	0.032777103	Decrease
EGF containing fibulin extracellular matrix protein 1	F6PVG3	0.033746242	Decrease
Histone H4	A0A3Q2IAY9	0.034130559	Increase
Complement C7	A0A3Q2I4S4	0.034130559	Decrease
Haptoglobin	F6XWM5	0.034758356	Increase
MIA SH3 domain containing	F6VJF5	0.039924238	Decrease
Complement factor D, EC 3.4.21.46 (Adipsin) (C3 convertase activator) (Properdin factor D)	A0A3Q2LBP6	0.039924238	Decrease
Hyaluronan binding protein 2	F6VZ73	0.039924238	Decrease
Cartilage intermediate layer protein	F7C2J3	0.039924238	Decrease
WD repeat-containing protein 91	A0A3Q2L5K2	0.039924238	Decrease
Dynamin GTPase, EC 3.6.5.5	A0A3Q2H544	0.039924238	Decrease
Complement factor B, EC 3.4.21.47 (C3/C5 convertase)	A0A3Q2IDD2	0.039924238	Decrease
RAB10, member RAS oncogene family	A0A3Q2H5J5	0.043202308	No change
Secreted phosphoprotein 24 (Secreted phosphoprotein 2)	F7DIX7	0.043202308	Increase

Complement C2, EC 3.4.21.43 (C3/C5 convertase)	F6PPQ0	0.043202308	Decrease
Heat shock 70kDa protein 1A	F7DW69	0.043202308	Decrease
Titin	A0A5F5PSR4	0.043202308	Increase
Folate hydrolase 1B	A0A3Q2HLX0	0.043202308	Decrease
Complement C1s	F7BQD6	0.043202308	Increase
Uncharacterized protein	H9GZV1	0.043202308	Increase
EGF containing fibulin extracellular matrix protein 2	A0A5F5PL54	0.04772571	Decrease

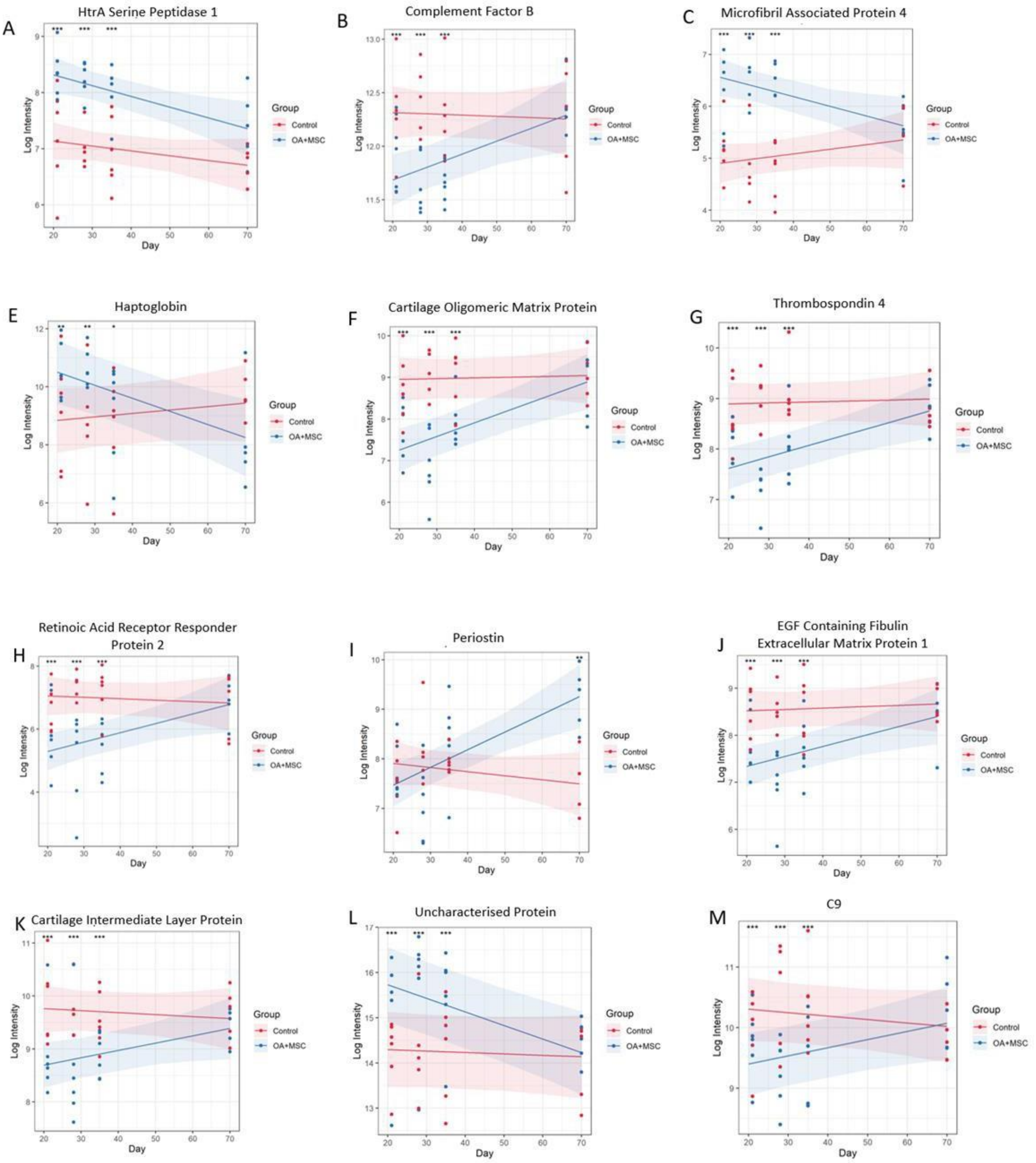
A
HtrA Serine Peptidase 1
B
Complement Factor B
C
Microfibril Associated Protein 4
E
Haptoglobin
F
Cartilage Oligomeric Matrix Protein
G
Thrombospondin 4
H
Retinoic Acid Receptor Responder Protein 2
I
Periostin
J
EGF Containing Fibulin Extracellular Matrix Protein 1
K
Cartilage Intermediate Layer Protein
L
Uncharacterised Protein
M
C9
Log Intensity
Day
Group
Control
OA+MSC

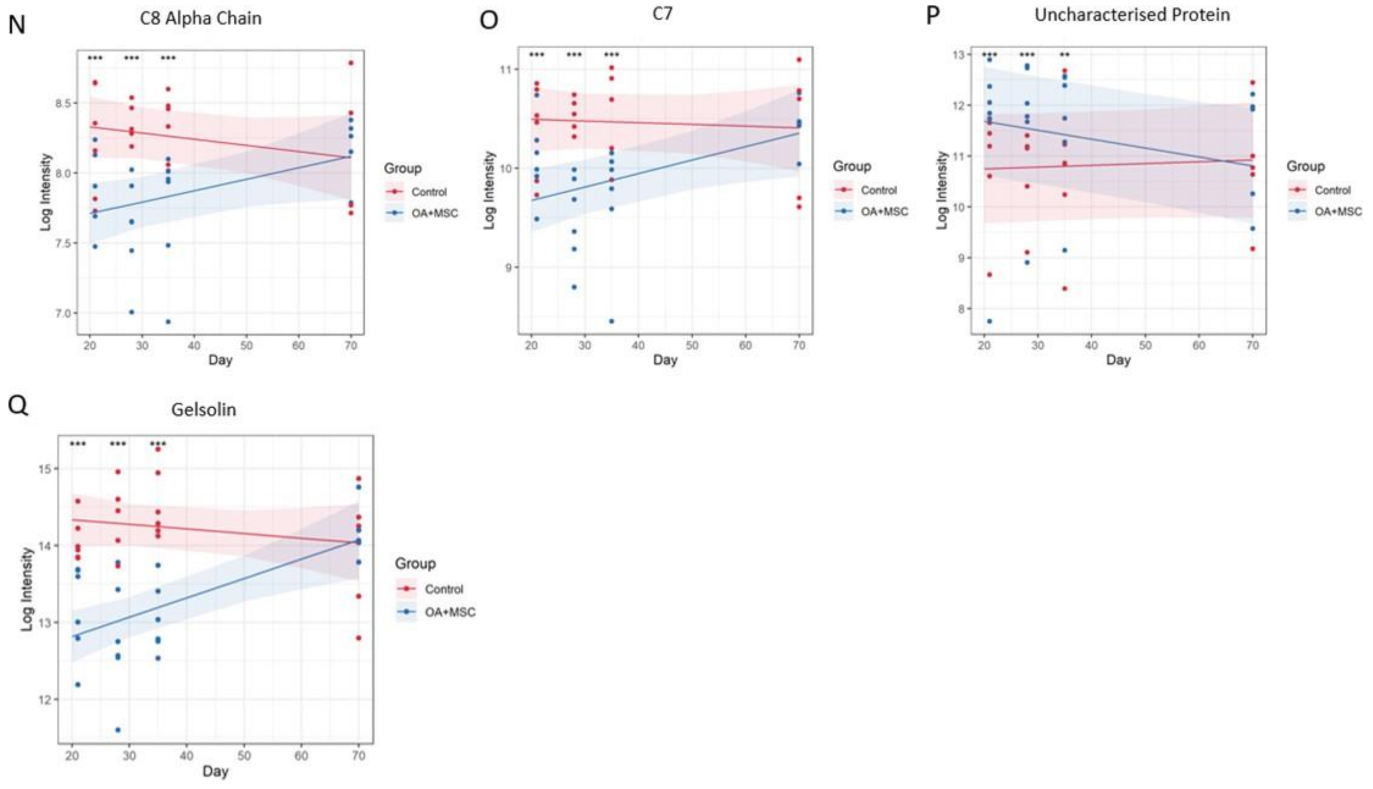

Figure 1. Differentially abundant proteins mapped to one of the three most significant functional enrichment pathway. Graphs demonstrating the results of significant ($p \leq 0.05$) proteins identified after a linear mixed model was applied to the experimental cohort, comparing protein expression between experimental group (treatment OA+MSCs (treatment) and control over time (day 21, 28, 35 and 70), visualizing the expression change longitudinally. Pairwise comparisons were conducted post linear mixed model application to compare control and OA +MSCs protein expression per time point. Significance level, as determined by the generated FDR corrected P Value is shown using ($p<0.05$, *; $p<0.01$ **: $p<0.001$, ***, $p<0.0001$, ****).
